# Secreted EMC10 inhibits muscle GLUT4 activity and glucose uptake in mice

**DOI:** 10.1016/j.jbc.2025.110296

**Published:** 2025-05-27

**Authors:** Shuoshuo Jin, Wei Wu, Shan Liu, Yahao Wang, Qi Huang, Kun He, Yunzhi Ni, Kuangyang Chen, Jinya Huang, Lijie Liu, Jiarong Dai, Chongwen Zhan, Xinru Wang, Yihui Guan, Matthias Blüher, Xuanchun Wang

**Affiliations:** 1 Department of Endocrinology, Huashan Hospital, Shanghai Medical College, Fudan University, Shanghai, China; 2 PET Center & Department of Nuclear Medicine, Huashan Hospital, Shanghai Medical College, Fudan University, Shanghai, China; 3 Department of Urology, Huashan Hospital, Shanghai Medical College, Fudan University, Shanghai, China; 4 Department of General Surgery, Huashan Hospital, Shanghai Medical College, Fudan University, Shanghai, China; 5 Helmholtz Institute for Metabolic, Obesity and Vascular Research (HI-MAG) of the Helmholtz Zentrum München at the University of Leipzig and University Hospital Leipzig, Germany; 6 Medical Department III–Endocrinology, Nephrology, Rheumatology, University of Leipzig Medical Center, Leipzig, Germany

**Keywords:** secreted EMC10, glucose uptake, skeletal muscle, GLUT4

## Abstract

Manipulation of glucose uptake plays a critical role in muscle glucose disposal. We have shown that the secreted isoform of endoplasmic reticulum membrane protein complex subunit 10 (scEMC10) impairs glucose tolerance in mice, and serum scEMC10 is positively associated with insulin resistance and hyperglycemia in humans. In this study, we attempt to investigate whether modulation of muscle glucose uptake implicates in the scEMC10-impacted glucose homeostasis. In mouse models, *Emc10* gene KO elevated, whereas recombinant scEMC10 treatment reduced muscle glucose uptake and GLUT4 expression. In myoblasts, scEMC10 inhibited both GLUT4 expression and membrane translocation and downregulated expression of genes associated with intracellular glucose metabolism. Mechanistically, scEMC10 suppressed the activation of muscle AMP-activated protein kinase and insulin signaling cascades. Inhibition of scEMC10 *via* a neutralizing antibody enhanced muscle glucose uptake in mice, in parallel with heightened GLUT4 expression and membrane translocation, which accounts for an improved whole-body glucose homeostasis. In conclusion, this work identifies scEMC10 as a novel suppressor of muscle glucose uptake and suggests inhibition of scEMC10 as a therapeutic strategy for type 2 diabetes.

Skeletal muscle plays an important role in maintaining blood glucose homeostasis and is responsible for ∼80% of postprandial glucose disposal (1). In the context of type 2 diabetes, insulin-stimulated glucose disposal of skeletal muscle is reduced by 50%, highlighting the importance of skeletal muscle in the pathogenesis of type 2 diabetes (2). Skeletal muscle glucose disposal is mainly fulfilled through three processes, including delivery, uptake, and intracellular metabolism of glucose, all of which are mediated *via* both insulin-dependent and -independent manners (3). Besides insulin, exercise is another well-known modulator of skeletal muscle glucose disposal, whose effect is largely independent of insulin since exercise-stimulated glucose uptake is well preserved regardless of insulin sensitivity (3, 4). Impacts of both insulin and exercise on skeletal muscle glucose uptake converge on the regulation of GLUT4, a highly abundant glucose transporter in skeletal muscle, which facilitates glucose uptake (3, 5). Insulin regulates GLUT4 transport *via* canonical Akt-AS160 and noncanonical Rac1-actin pathways, whereas exercise-elicited GLUT4 translocation mainly involves the activation of AMP-activated protein kinase (AMPK) signaling pathway (1). Once activated, AMPK phosphorylates downstream target TBC1D1, promoting GLUT4 translocation in skeletal muscle (6). Beyond GLUT4 translocation, AMPK signaling is also required for the regulation of GLUT4 expression, which involves the coordination of a line of transcriptional factors and cofactors, including myocyte enhancer factor 2 (MEF2), HDAC5, PGC-1α, KLF15, and MyoD (7).

Endoplasmic reticulum (ER) membrane protein complex subunit 10 (EMC10) consists of two isoforms because of differential splicing of the *EMC10* gene, including a membrane-bound isoform (mEMC10) localized at the lumen of ER and a secreted isoform (scEMC10) present in circulation (8, 9). scEMC10 has been associated with hepatic ER stress and steatosis (10), glioma tumorigenesis and angiogenesis (11), as well as myocardial neovascularization postinfarction (12). We have identified scEMC10 to play a crucial role in glucose homeostasis, where overexpression of scEMC10 impairs, whereas antibody neutralization of circulating scEMC10 enhances glucose tolerance in mice, and serum scEMC10 is positively associated with insulin resistance and hyperglycemia in humans (13). The underlying mechanism, however, remains largely unknown.

In this study, we attempt to address whether scEMC10-modulated glucose homeostasis is fulfilled through its impact on muscle glucose disposal. By employing experiments on cell culture and mouse models, we identified that scEMC10 suppressed muscle glucose uptake *via* inhibition of both GLUT4 translocation and expression, which involve multiple signaling pathways, and inhibition of scEMC10 may be a therapeutic strategy for type 2 diabetes *via* enhancement of muscle glucose uptake.

## Results

### scEMC10 suppresses glucose uptake *via* inhibition of both GLUT4 expression and translocation

**Figure 1:**
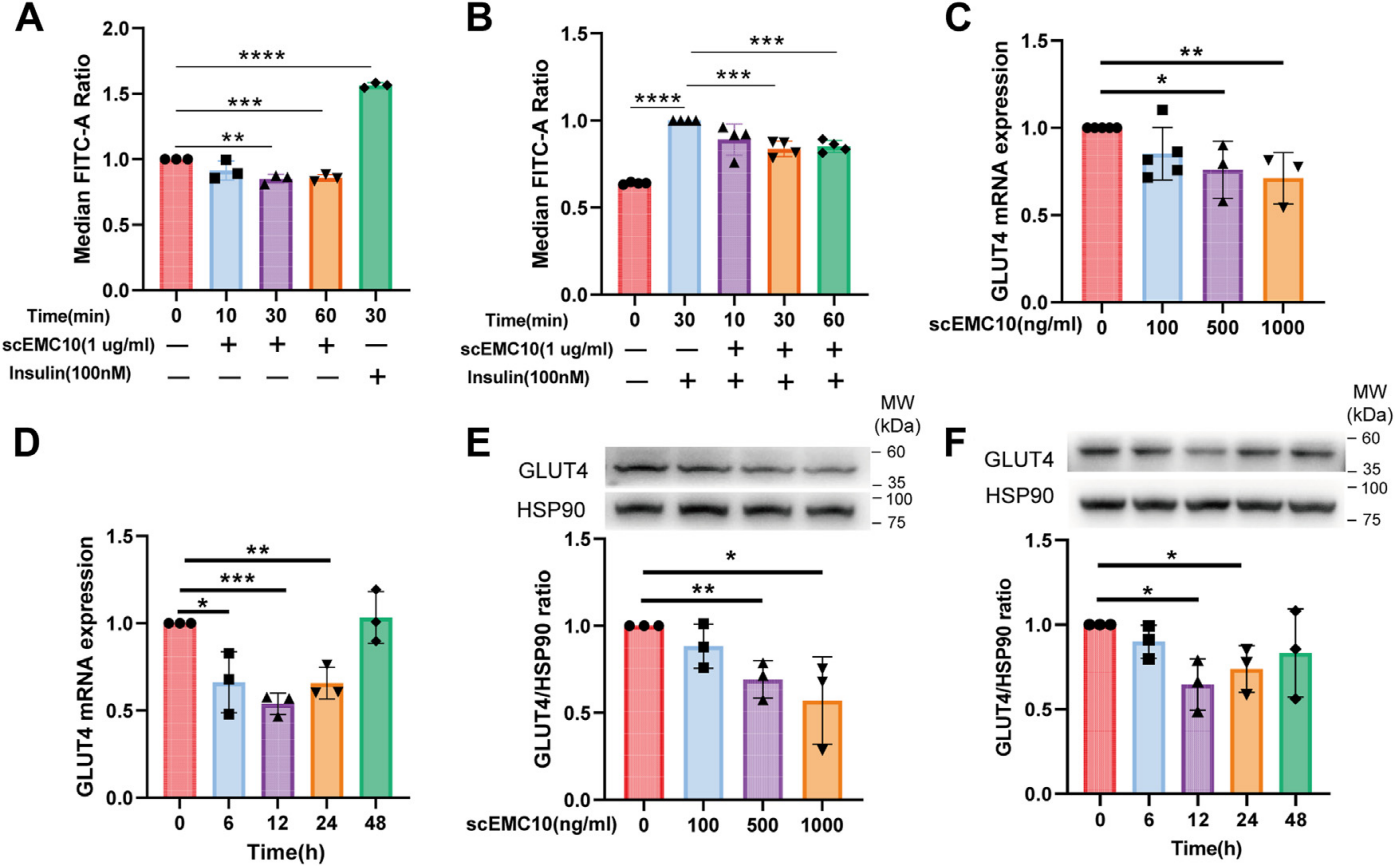
**scEMC10 inhibits glucose uptake and GLUT4 expression in skeletal muscle cells**. *A*, L6-GLUT4myc myotubes were treated with 1 μg/ml recombinant scEMC10 protein for various times as indicated in the presence of 100 μmol/l 2-NBDG. 2-NBDG uptake into the myotubes was measured at the FITC channel by a flow cytometer. Meanwhile, 2-NBDG uptake stimulated by 100 nM insulin for 30 min was used as a positive control. *B*, L6-GLUT4myc myotubes were treated with 100 nM insulin in combination with or without 1 μg/ml recombinant scEMC10 protein for the times as indicated. 2-NBDG uptake into the myotubes was measured. *C–F*, C2C12 myotubes were treated with recombinant scEMC10 protein at different concentrations as indicated for 12 h, and then the levels of GLUT4 mRNA (*C*) and protein (*E*) were determined by quantitative PCR and Western blot, respectively. C2C12 myotubes were treated with 1 μg/ml recombinant scEMC10 protein for various times as indicated, and then the levels of GLUT4 mRNA (*D*) and protein (*F*) were determined by quantitative PCR and Western blot, respectively. All cell culture experiments were repeated three to four times. All data are presented with means ± SD. In experiments where samples were prepared in several batches, the control conditions were set at 100% in individual experiments and therefore had no error estimates. Statistical analyses were performed using unpaired two-tailed Student's *t* test, and significant differences were indicated with *p* values. ∗*p* < 0.05, ∗∗*p* < 0.01, ∗∗∗*p* < 0.001, and ∗∗∗∗*p* < 0.0001. 2-NBDG, 2-[*N*-(7-nitrobenz-2-oxa-1,3-diazol-4-yl) amino]-2-deoxy-d-glucose; scEMC10, secreted isoform of endoplasmic reticulum membrane protein complex subunit 10.

To investigate the impact of scEMC10 on muscle glucose uptake, 2-NBDG (2-[*N*-(7-nitrobenz-2-oxa-1,3-diazol-4-yl) amino]-2-deoxy-d-glucose), a fluorescent deoxyglucose analog, was employed to trace glucose uptake into L6-GLUT4myc myotubes. The optimal concentration of 2-NBDG was determined at 100 μM (Fig. S1*A*). As expected, insulin robustly increased the glucose uptake into the myotubes in a dose-dependent manner (Fig. S1B). scEMC10 significantly decreased both basal and insulin-stimulated glucose uptake into L6-GLUT4myc myotubes (Fig. 1, *A* and *B*). To explore how scEMC10 regulates glucose uptake, GLUT4 expression was assessed in skeletal muscle cells. It was observed that levels of both GLUT4 mRNA and protein were significantly reduced by recombinant scEMC10 protein in time- and dose-dependent manners in C2C12 myotubes (Fig. 1, *C–F*).

**Figure 2:**
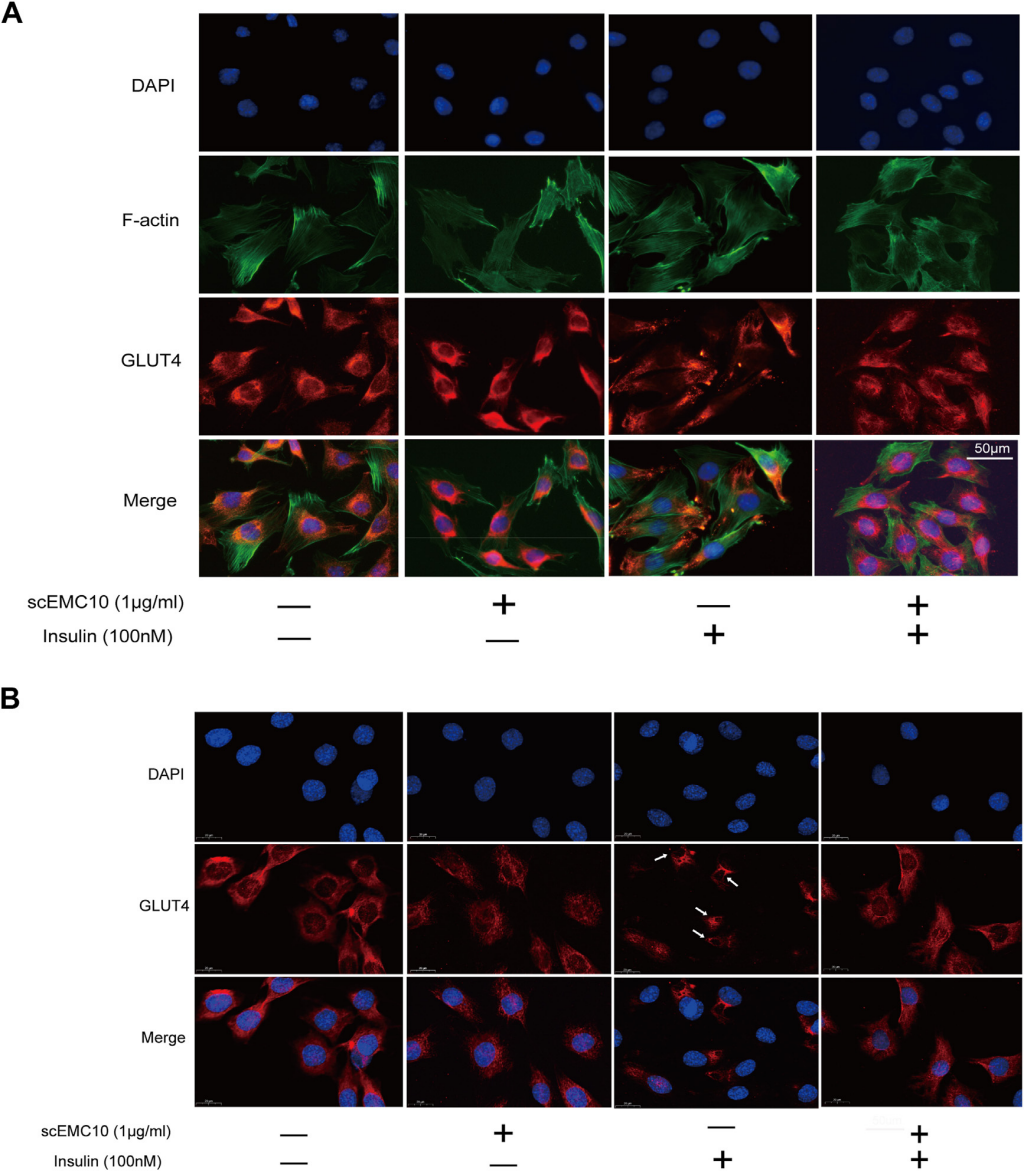
**scEMC10 suppresses GLUT4 translocation in L6-GLUT4myc myoblasts**. *A* and *B*, L6-GLUT4myc myoblasts grown on slides were treated with 1 μg/ml recombinant scEMC10 protein, 100 nmol/l insulin, or scEMC10 in combination with insulin for 30 min, respectively, and then GLUT4 distribution was assessed using immunofluorescent staining in either permeabilized (*A*) or nonpermeabilized (*B*) cells. In *B*, *white arrows* indicate ring-like staining of GLUT4. Scale bar represents 50 μm (*A*) and 20 μm (*B*). All cell culture experiments were repeated three to four times. scEMC10, secreted isoform of endoplasmic reticulum membrane protein complex subunit 10.

Translocation of GLUT4 from cytosol to plasma membrane is a prerequisite for both insulin- and exercise-induced glucose uptake (7). Here, scEMC10-modulated GLUT4 translocation was investigated in either permeabilized or nonpermeabilized L6-GLUT4myc myoblasts using immunofluorescent staining. We observed that GLUT4 stayed around the nucleus in the basal state, scEMC10 treatment condensed GLUT4 in the perinucleus, whereas insulin drove GLUT4 to move away from the nucleus and colocalize with filamentous actin in permeabilized L6-GLUT4myc myoblasts, indicating membrane translocation of GLUT4 (Fig. 2*A*). scEMC10 prevented the translocation of GLUT4 induced by insulin (Fig. 2*A*). In the nonpermeabilized myoblasts, GLUT4 stayed in the perinuclear region at baseline, whereas insulin stimulation resulted in its ring-like staining (Fig. 2*B*, as indicated by *white arrows*), a sign of its membrane localization. scEMC10 treatment restricted GLUT4 to the perinuclear region again in these insulin-stimulated cells (Fig. 2*B*), similar to the observation in permeabilized cells (Fig. 2*A*). In sum, here we provided comprehensive evidence to assess insulin and scEMC10-modulated GLUT4 translocation, which was judged by the distance from GLUT4 to the nucleus and the colocalization of GLUT4 with filamentous actin in permeabilized L6-GLUT4myc myoblasts, in combination with membrane localization of GLUT4 in the nonpermeabilized myoblasts.

Taken together, these data indicated that scEMC10 suppressed both basal and insulin-stimulated glucose uptake into skeletal muscle cells *via* inhibition of both expression and membrane translocation of GLUT4.

### scEMC10 suppresses GLUT4 expression and glucose uptake into mouse skeletal muscle tissue

**Figure 3:**
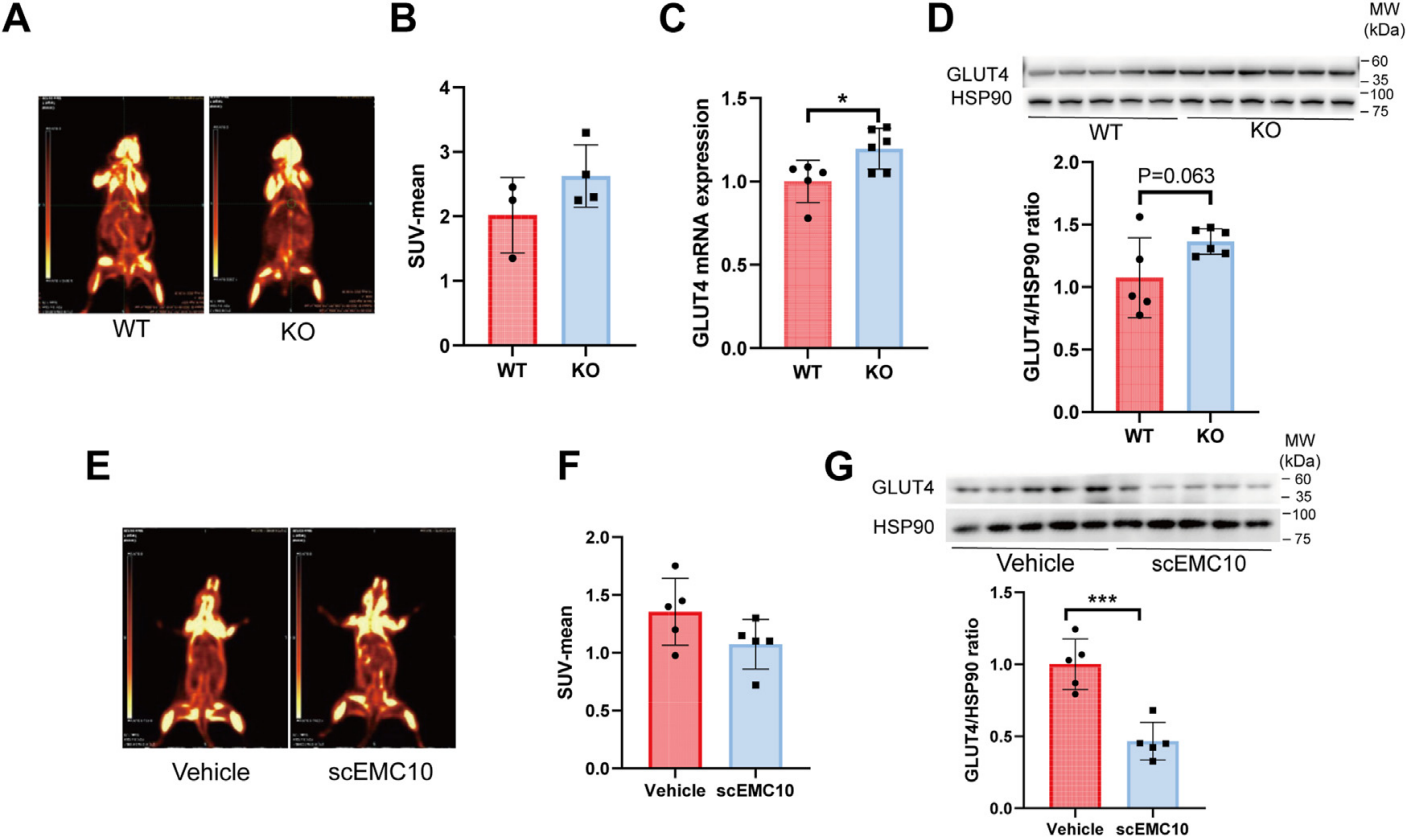
**scEMC10 inhibits muscle glucose uptake and GLUT4 expression in mice.***A* and *B*, representative images (*A*) and quantifications (*B*) of ^18^F-FDG uptake into gastrocnemius determined using PET–CT scan in *Emc10* KO (n = 3) or WT mice (n = 4) fed an HFD. *C* and *D*, levels of GLUT4 mRNA (*C*) and protein (*D*) were determined in gastrocnemius tissue of the mice (n = 5–6) indicated by quantitative PCR and Western blot, respectively. *E* and *F*, representative images (*E*) and quantifications (*F*) of ^18^F-FDG uptake into gastrocnemius determined using PET–CT scan in mice intraperitoneally injected with recombinant mouse scEMC10 protein (6 mg per kg body weight) (n = 5) or vehicle (human IgG1 Fc) (n = 6). *G*, GLUT4 protein levels were measured in gastrocnemius tissue of mice intraperitoneally injected with recombinant mouse scEMC10 protein (n = 5) or vehicle (n = 5) by Western blot. All data are presented with means ± SD. Statistical analyses were performed using unpaired two-tailed Student's *t* test, and significant differences were indicated with *p* values. ∗*p* < 0.05, ∗∗∗*p* < 0.001. ^18^F-FDG, ^18^F-fluorodeoxyglucose; CT, computed tomography; HFD, high-fat diet; scEMC10, secreted isoform of endoplasmic reticulum membrane protein complex subunit 10.

We have shown that *Emc10* KO improves diet-induced glucose intolerance in mice (13). To explore whether modulation of muscle glucose disposal is involved in the process, we measured glucose uptake and GLUT4 expression in skeletal muscle tissue of mice. PET–computed tomography (CT) analysis indicated that glucose uptake into gastrocnemius trended higher in *Emc10* KO mice than WT mice under the condition of high-fat diet (HFD) feeding (Fig. 3, *A* and *B*), which can be accounted for by increased levels of both GLUT4 mRNA and protein in KO skeletal muscle tissue (Fig. 3, *C* and *D*). Conversely, intraperitoneal injection of recombinant scEMC10 protein reduced glucose uptake into mouse gastrocnemius, concomitant with a decrease of GLUT4 protein in the muscle tissue, when compared with vehicle (Fig. 3, *E–G*). These data, together with our previous observation that overexpression of scEMC10 impairs glucose tolerance in mice (13), suggest that scEMC10 acts as a novel suppressor of muscle glucose uptake, consequentially promoting hyperglycemia.

### scEMC10 negatively modulates AMPK and insulin signaling pathways in skeletal muscle

**Figure 4:**
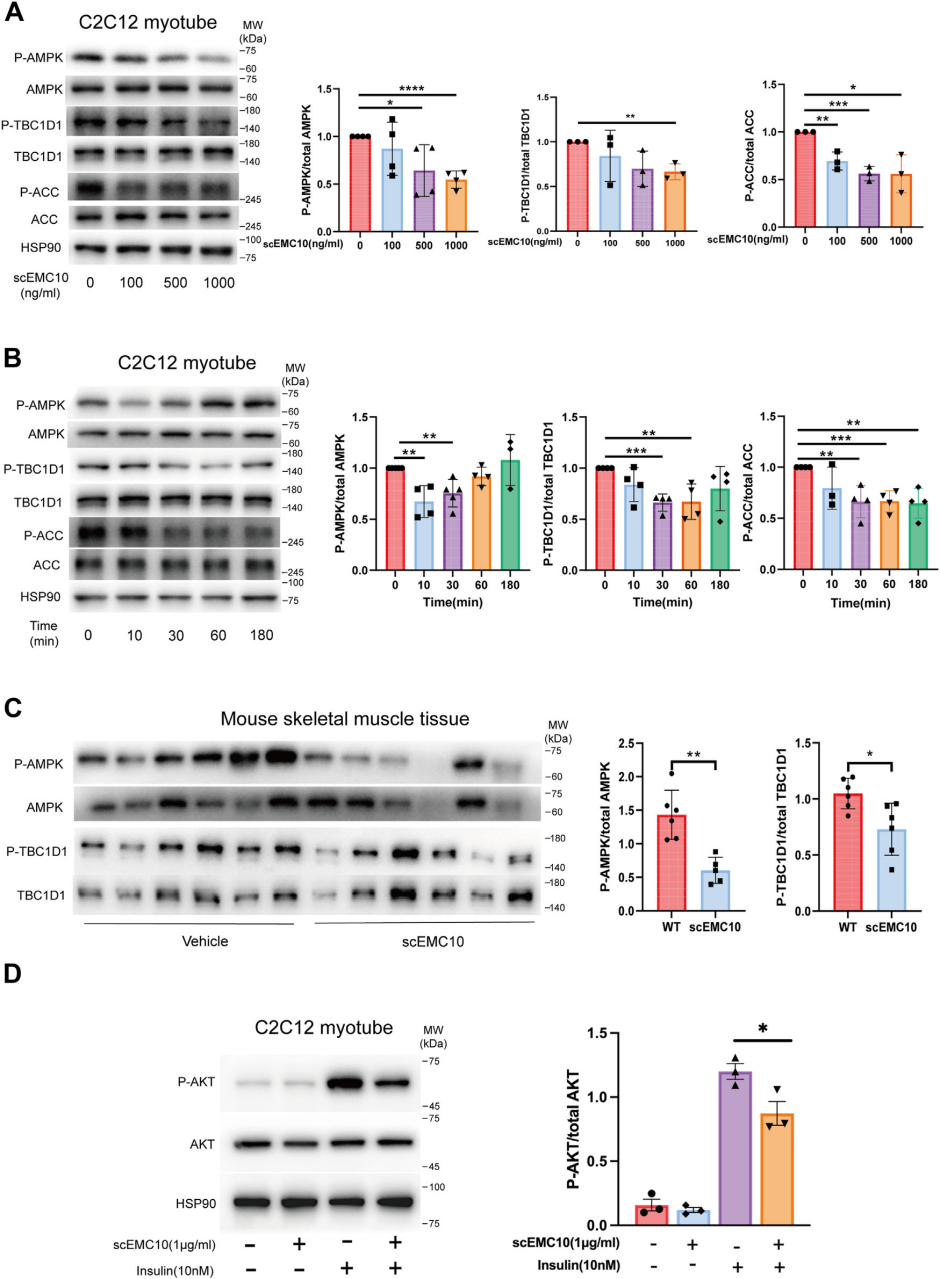
**scEMC10 negatively modulates muscle AMPK and insulin signaling pathways**. *A* and *B*, C2C12 myotubes were treated with recombinant mouse scEMC10 protein at different concentrations as indicated for 10 min (*A*) or with 1 μg/ml recombinant scEMC10 protein for various times as indicated (*B*), and then phosphorylated AMPK, AMPK, phosphorylated TBC1D1, TBC1D1, phosphorylated ACC, ACC, and HSP90 were determined using Western blot. *C*, C57BL/6J mice were intraperitoneally injected with recombinant mouse scEMC10 protein (6 mg per kg body weight) (n = 6) or vehicle (human IgG1 Fc) (n = 6) for 30 min, and then phosphorylated AMPK, AMPK, phosphorylated TBC1D1, and TBC1D1 were determined in gastrocnemius tissue using Western blot. All cell culture experiments were repeated four times. *D*, C2C12 myotubes were stimulated with either 10 nmol/l insulin for 10 min, 1 μg/ml recombinant scEMC10 for 6 h, or insulin for 10 min following 6 h of incubation with scEMC10, and then phosphorylated AKT and AKT were determined using Western blot. All cell culture experiments were repeated three to four times. All data are presented with means ± SD. In experiments where samples were prepared in several batches, the control conditions were set at 100% in individual experiments and therefore had no error estimates. Statistical analyses were performed using unpaired two-tailed Student's *t* test, and significant differences were indicated with *p* values. ∗*p* < 0.05, ∗∗*p* < 0.01, ∗∗∗*p* < 0.001, and ∗∗∗∗*p* < 0.0001. AMPK, AMP-activated protein kinase; scEMC10, secreted isoform of endoplasmic reticulum membrane protein complex subunit 10.

In skeletal muscle, multiple signaling pathways are involved in glucose uptake *via* regulation of GLUT4 activity, including AMPK and insulin signaling pathways (7, 14). To dissect the underlying mechanism of scEMC10-regulated glucose uptake, we first investigated its impact on muscle AMPK signaling. In C2C12 myotubes, we identified a suppressive role for scEMC10 in AMPK signaling, evidenced by significant reductions in phosphorylation of AMPK and its downstream targets—TBC1D1 and ACC (Fig. 4, *A* and *B*). scEMC10 also significantly decreased phosphorylated AMPK and TBC1D1 in mouse skeletal muscle tissue (Fig. 4*C*). We next investigated whether scEMC10 exerted an impact on muscle insulin signaling pathway. We observed that scEMC10 significantly suppressed insulin-induced Akt phosphorylation in C2C12 myotubes (Fig. 4*D*). Taken together, these data suggest that scEMC10 suppresses GLUT4 activity and glucose uptake in skeletal muscle through AMPK and insulin signaling cascades.

### Inhibition of scEMC10 enhances glucose uptake in mouse skeletal muscle

**Figure 5:**
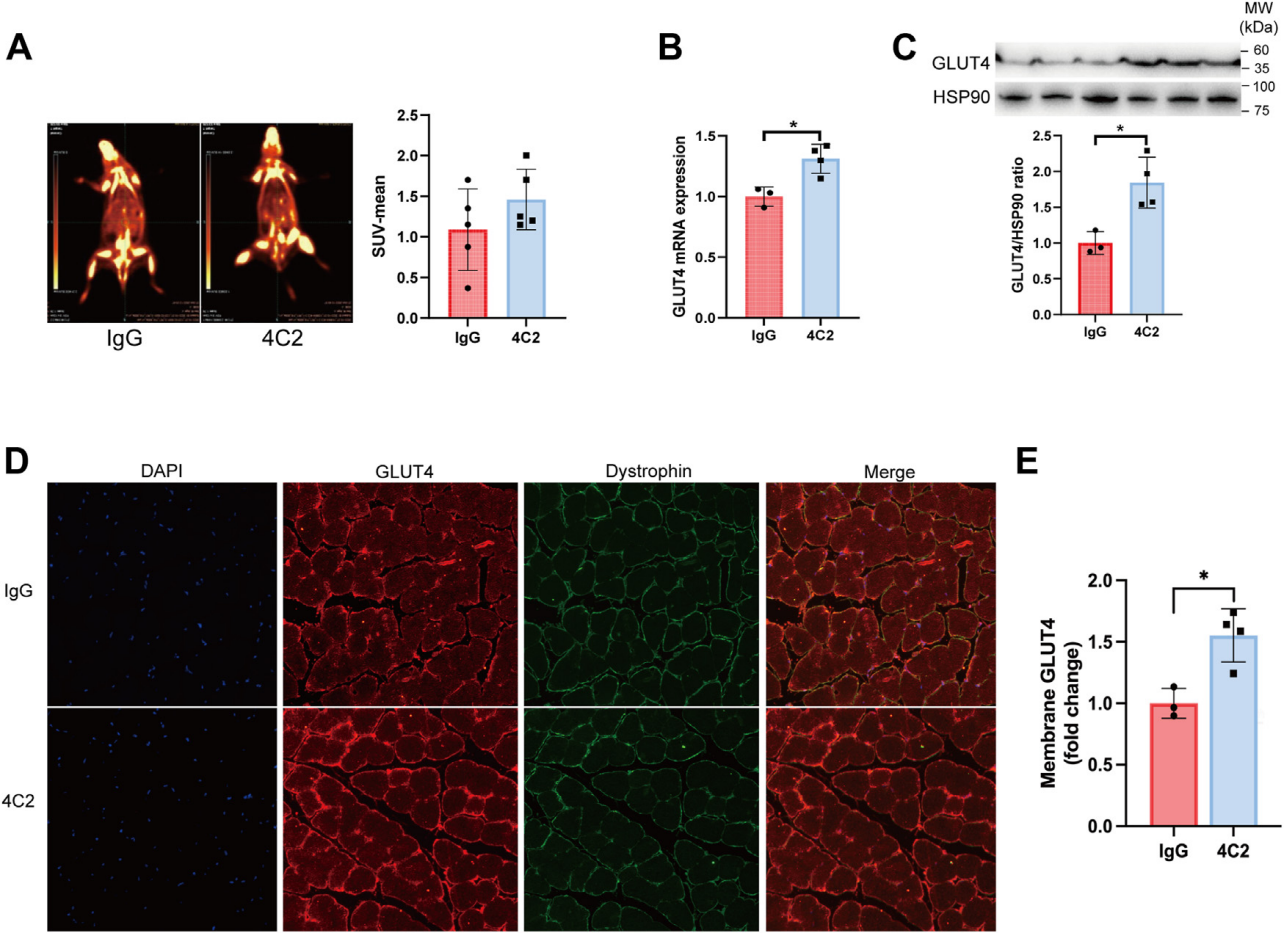
**Antibody neutralization of scEMC10 enhances GLUT4 expression and glucose uptake in mouse skeletal muscle**. *A*, representative images and quantifications of ^18^F-FDG uptake into gastrocnemius determined using PET–CT scan in HFD-fed mice intraperitoneally injected with scEMC10-neutralizing antibody 4C2 (3 mg per kg body weight) (n = 5) or control IgG (n = 5). *B* and *C*, levels of GLUT4 mRNA (*B*) and protein (*C*) were determined in gastrocnemius tissue of the mice (n = 3–4) indicated by quantitative PCR and Western blot, respectively. *D*, immunofluorescent staining of GLUT4 and dystrophin in gastrocnemius tissue of the mice. *E*, quantitative analysis of membraneous GLUT4 immunofluorescence normalized to dystrophin in gastrocnemius muscle tissue of mice using ImageJ (n = 3–4). All data are presented with means ± SD. Statistical analyses were performed using unpaired two-tailed Student's *t* test, and significant differences were indicated with *p* values. ∗*p* < 0.05. CT, computed tomography; ^18^F-FDG, ^18^F-fluorodeoxyglucose; HFD, high-fat diet; scEMC10, secreted isoform of endoplasmic reticulum membrane protein complex subunit 10.

We previously identified that a monoclonal antibody termed as 4C2, which efficiently neutralized circulating scEMC10, enhanced glucose tolerance in HFD-fed mice, suggesting that inhibition of scEMC10 may have a therapeutic potential for the treatment of type 2 diabetes (13). The underlying mechanism, however, remains elusive. In this study, we observed that the scEMC10-neutralizing antibody trended to elevate glucose uptake into mouse skeletal muscle by PET–CT analysis (Fig. 5*A*). This was contributed by increased muscle expression of both GLUT4 mRNA and protein in mice treated with the neutralizing antibody as compared with mice with control immunoglobulin G (IgG) (Fig. 5, *B* and *C*). Immunofluorescence analysis indicated 55% greater membraneous GLUT4 in skeletal muscle tissue of the antibody-treated mice than that of control mice (Fig. 5, *D* and *E*).

**Figure 6:**
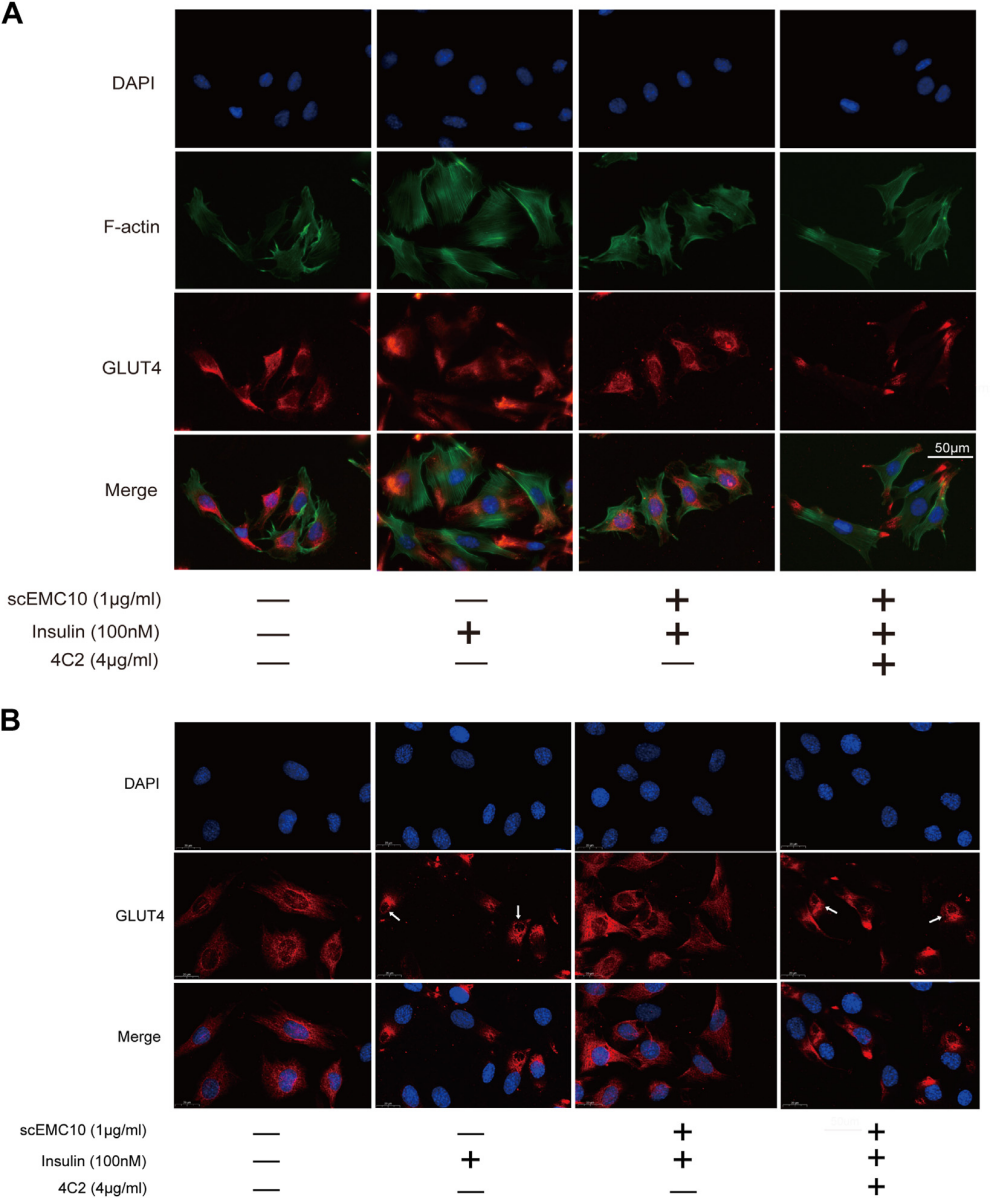
**Inhibition of scEMC10 reverses its inhibitory effect on GLUT4 translocation in L6-GLUT4myc myoblasts**. *A* and *B*, L6-GLUT4myc myoblasts grown on slides were treated with 100 nmol/l insulin, 1 μg/ml recombinant scEMC10 protein, and 4 μg/ml scEMC10-neutralizing antibody 4C2 as indicated for 30 min, respectively, and then GLUT4 distribution was assessed using immunofluorescent staining in either permeabilized (*A*) or nonpermeabilized (*B*) cells. In *B*, *white arrows* indicate ring-like staining of GLUT4. Scale bar represents 50 μm (*A*) and 20 μm (*B*). All cell culture experiments were repeated three to four times. scEMC10, secreted isoform of endoplasmic reticulum membrane protein complex subunit 10.

To further confirm the effect of scEMC10 inhibition on GLUT4 translocation, we performed *in vitro* experiments where scEMC10-modulated GLUT4 translocation was intervened with the neutralizing antibody in L6-GLUT4myc myoblasts (Fig. 6). Consistent with the observation in Figure 2, scEMC10 repressed the insulin-induced GLUT4 translocation (Fig. 6, *A* and *B*). Its neutralizing antibody, however, released its brake on the effect of insulin in either permeabilized or nonpermeabilized myoblasts (Fig. 6, *A* and *B*).

These data support a glucose-lowering effect of scEMC10 inhibition *via* enhancement of glucose uptake in skeletal muscle.

### scEMC10 regulates expression of transcriptional factors of GLUT4 in muscle cells

**Figure 7:**
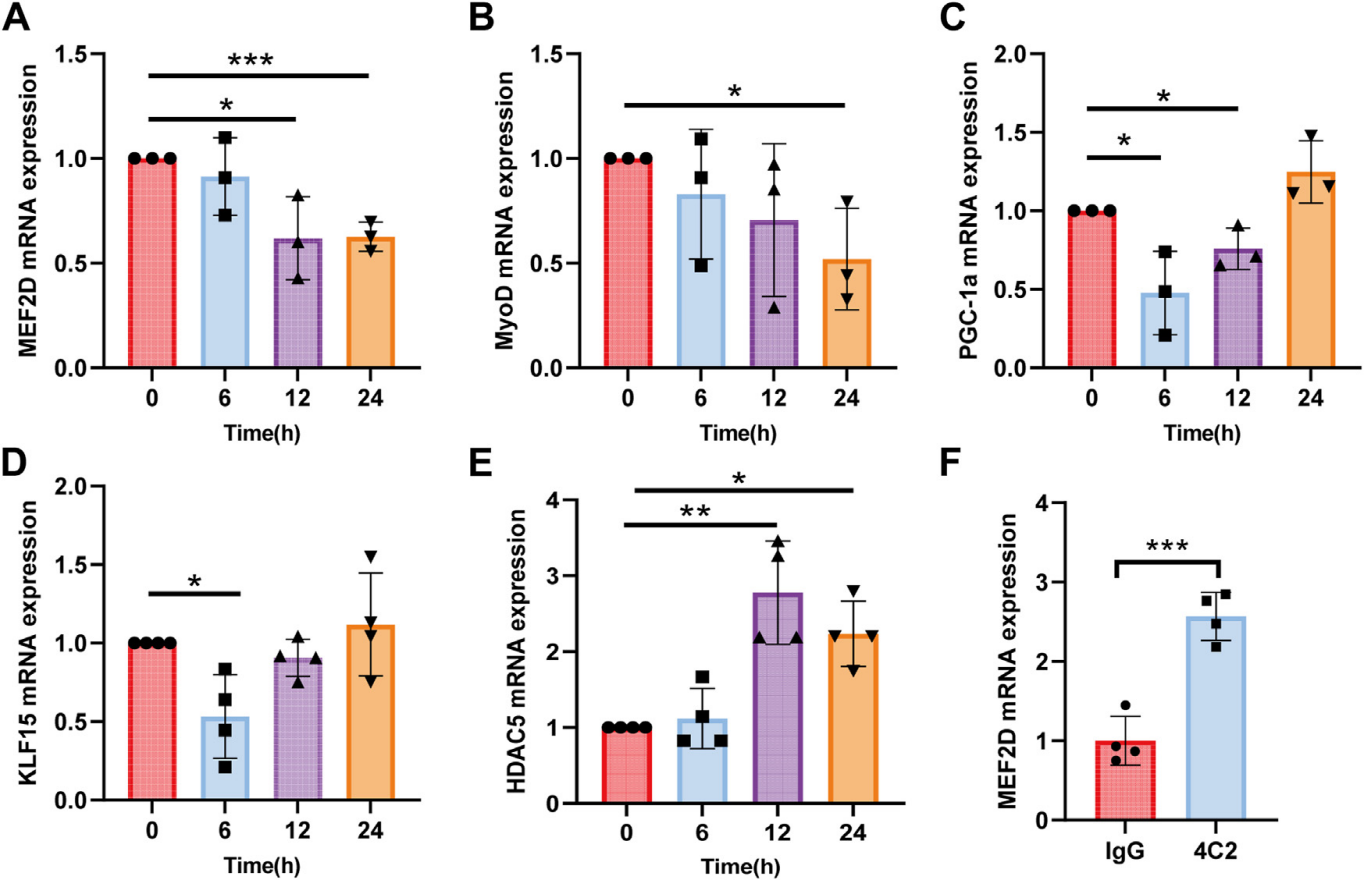
**scEMC10 regulates expression of transcriptional factors of GLUT4 in C2C12 myotubes**. *A–E*, C2C12 myotubes were treated with 1 μg/ml recombinant scEMC10 protein for various times as indicated, and then mRNA levels of MEF2D (*A*), MyoD (*B*), PGC-1α (*C*), KLF15 (*D*), and HDAC5 (*E*) were measured using quantitative PCR. *F*, MEF2D mRNA levels were assessed in gastrocnemius tissue of mice intraperitoneally injected with scEMC10-neutralizing antibody 4C2 (3 mg per kg body weight) (n = 4) or control IgG (n = 4). All cell culture experiments were repeated three to four times. All data are presented with means ± SD. In experiments where samples were prepared in several batches, the control conditions were set at 100% in individual experiments and therefore had no error estimates. Statistical analyses were performed using unpaired two-tailed Student's *t* test, and significant differences were indicated with *p* values. ∗*p* < 0.05, ∗∗*p* < 0.01, and ∗∗∗*p* < 0.001. scEMC10, secreted isoform of endoplasmic reticulum membrane protein complex subunit 10.

In addition to GLUT4 translocation, glucose uptake also relies on GLUT4 expression in skeletal muscle. Aforementioned data demonstrate that scEMC10 exerts a regulatory role in muscle GLUT4 expression. A battery of transcriptional factors has been implicated in the regulation of GLUT4 expression, in which the MEF2 family of transcription factors play a pivotal role (7). We observed that recombinant scEMC10 protein significantly decreased mRNA levels of MEF2D in C2C12 myotubes (Fig. 7*A*), in parallel with significant reductions in gene expression of MyoD, PGC-1α, and KLF15 (Fig. 7, *B–D*), all of which have been shown to synergize with MEF2 in activation of the GLUT4 promoter (15, 16, 17). Conversely, scEMC10 significantly increased mRNA levels of HDAC5 (Fig. 7*E*), a transcriptional repressor of MEF2 (18). In mice, antibody neutralization of circulating scEMC10 led to a significant increase in mRNA levels of MEF2D in skeletal muscle tissue (Fig. 7*F*). These data provide a mechanistic insight into scEMC10-modulated GLUT4 expression in skeletal muscle.

### scEMC10 downregulates the expression of genes associated with intracellular glucose metabolism

**Figure 8:**
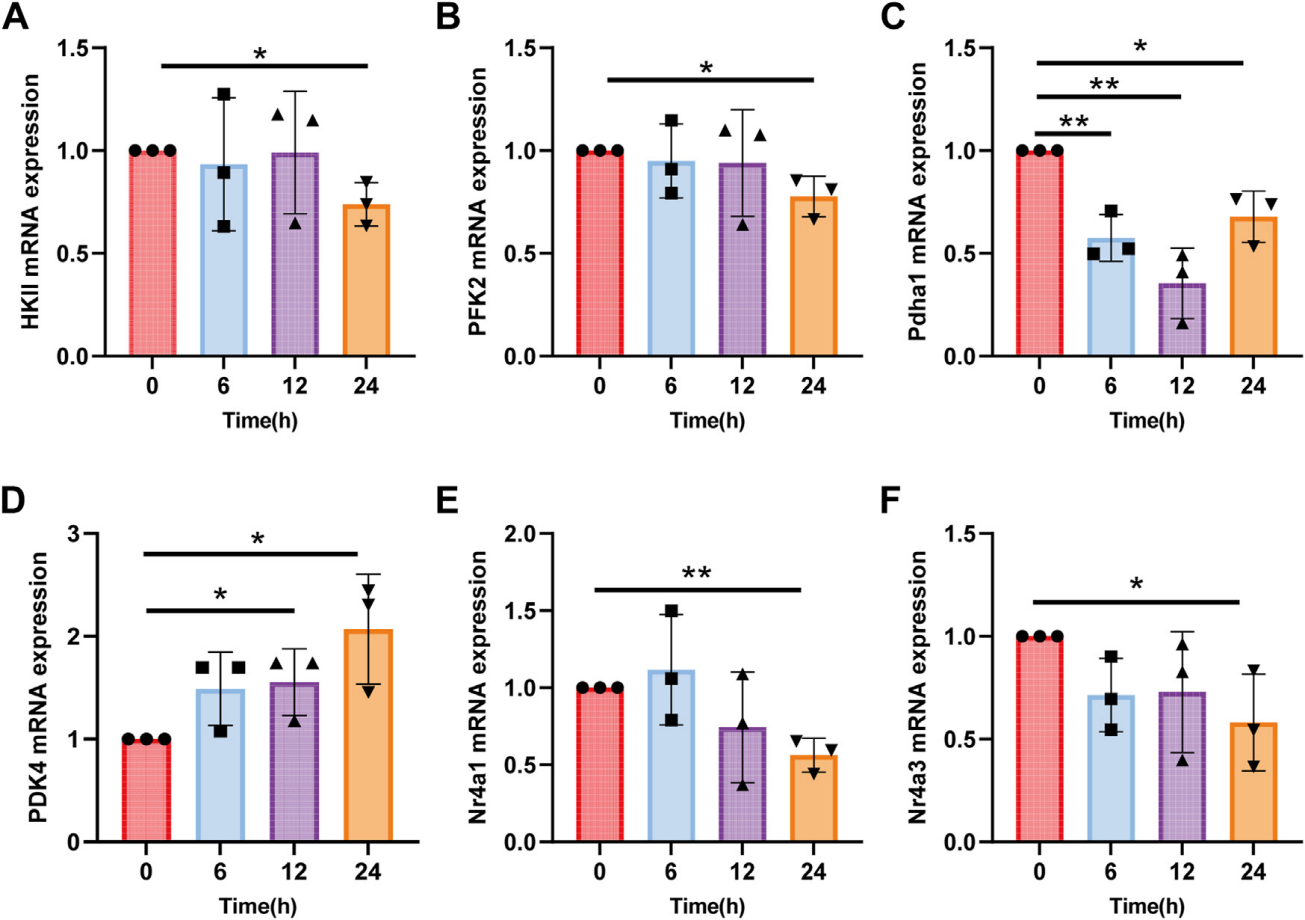
**scEMC10 modulates expression of genes associated with intercellular glucose metabolism in C2C12 myotubes**. *A–F*, C2C12 myotubes were treated with 1 μg/ml recombinant scEMC10 protein for various times as indicated, and then mRNA levels of HKII (*A*), PFK2 (phosphofructokinase 2) (*B*), Pdha1 (*C*), PDK4 (pyruvate dehydrogenase kinase 4) (*D*), Nr4a1 (*E*), and Nr4a3 (*F*) were measured using quantitative PCR. All cell culture experiments were repeated three times. All data are presented with means ± SD. In experiments where samples were prepared in several batches, the control conditions were set at 100% in individual experiments and therefore had no error estimates. Statistical analyses were performed using unpaired two-tailed Student's *t* test, and significant differences were indicated with *p* values. ∗*p* < 0.05, ∗∗*p* < 0.01. scEMC10, secreted isoform of endoplasmic reticulum membrane protein complex subunit 10.

Once transported into cells, glucose will undergo a metabolic flux, which is required for the maintenance of the infusion gradient to facilitate further glucose transport (3). To investigate whether scEMC10 impacts the metabolic flux, we assessed expression of genes encoding key enzymes involved in skeletal muscle glucose metabolism. In C2C12 myotubes, recombinant scEMC10 protein significantly decreased mRNA levels of hexokinase II (Fig. 8*A*), which catalyzes the phosphorylation of glucose to form glucose-6-phosphate, phosphofructokinase 2 (Fig. 8*B*), which controls glycolytic flux, and pyruvate dehydrogenase (Pdha1) (Fig. 8*C*), an enzyme guiding glucose flux to the tricarboxylic acid cycle (19). In accordance with the downregulation of Pdha1, scEMC10 significantly upregulated gene expression of pyruvate dehydrogenase kinase 4 (Fig. 8*D*), an inhibitory kinase of Pdha1 (20). In addition, the orphan nuclear receptors, Nr4a1 and Nr4a3, both of which have been linked to positively modulate muscle glucose metabolism, including increases in activities of GLUT4, hexokinase, and phosphofructokinase (21, 22), were identified to be downregulated by recombinant scEMC10 protein in C2C12 myotubes (Fig. 8*E* and *F*). These transcriptional data provide evidence that in parallel with the inhibition of glucose uptake, scEMC10 downregulates intracellular glucose metabolism in skeletal muscle.

## Discussion

Manipulation of glucose transporters plays a pivotal role in both muscle glucose disposal and whole-body glucose homeostasis. In patients with type 2 diabetes, both GLUT4 membrane translocation and expression are reduced in skeletal muscle, and exercise enhances GLUT4 translocation regardless of insulin sensitivity (23, 24). In this study, we identified that scEMC10 exerted inhibitory roles in the regulation of both GLUT4 expression and translocation. This is consistent with our previous observations showing that heightened circulatory levels of scEMC10 impairs glucose tolerance in mice, and serum EMC10 (scEMC10) is positively associated with blood glucose in humans (13). The findings of this study suggest that *via* modulation of muscle GLUT4, scEMC10 is implicated in the dysregulation of blood glucose in type 2 diabetes.

The regulatory actions of scEMC10 on GLUT4 involve both AMPK and insulin signaling cascades. scEMC10 exerts a direct, suppressive role in activation of the muscle AMPK pathway, which is required for exercise-stimulated muscle glucose uptake. Moreover, the effect of AMPK signaling on glucose uptake has been proven to be additive and insulin independent (7, 25). The mechanism underlying the modulation of the AMPK pathway by scEMC10 remains unknown. The pathway has been shown to be activated by an increase in cytosolic Ca^2+^ concentration (25, 26). This raises the possibility that scEMC10 exerts a regulatory role in cytosolic Ca^2+^, which warrants further exploration. Besides its involvement in contraction-induced GLUT4 translocation, AMPK also promotes GLUT4 expression *via* a transcriptional machinery (7). We demonstrated that scEMC10 modulates expression of multiple GLUT4 transcriptional factors, which is most likely attributed to its impact on AMPK signaling.

scEMC10 suppresses both insulin-stimulated GLUT4 translocation in myoblasts and glucose uptake into myotubes, in parallel with the inhibition of Akt-mediated insulin signaling. In humans, serum scEMC10 has been positively associated with insulin resistance (13). These findings implicate the induction of insulin resistance as another mechanism underlying the scEMC10-modulated muscle glucose uptake. scEMC10, in itself, is not capable of impacting muscle Akt activity either *in vitro* or *in vivo* (data not shown), suggesting scEMC10 plays an indirect role in modulating muscle insulin signaling.

We have demonstrated that serum scEMC10 is positively associated with blood glucose in humans, and an scEMC10-neutralizing antibody lowers blood glucose in mice (13). Here, we present evidence that the neutralizing antibody enhances muscle glucose uptake in mice *via* promoting both GLUT4 translocation and expression, in accordance with its glucose-lowering effect. These findings suggest that scEMC10 inhibition has therapeutic potential in type 2 diabetes *via* the enhancement of muscle glucose disposal. Given the suppressive role of scEMC10 in insulin-stimulated GLUT4 translocation and glucose uptake, scEMC10 inhibition will potentiate the stimulatory effect of insulin on muscle glucose disposal, thus lowering postprandial blood glucose. Although exercise-stimulated glucose transport has been shown to be intact in type 2 diabetes (24, 27), considering that the capacity of exercise is decreased in type 2 diabetes (28), the scEMC10-neutralizing antibody, serving as an exercise-mimetic agent, will also heighten muscle glucose disposal *via* derepression of the AMPK signaling. Although multiple exercise mimetics have been developed, such as GW501516, a peroxisome proliferator–activated receptor δ agonist (29), and nicotinamide riboside, an sirtuin 1 agonist (30), none of these have been proven successful in clinical use because of manifold unfavorable effects (1). Given scEMC10 as a suppressor or of AMPK, scEMC10 inhibitors will act as AMPK activators, similar to other well-known activators, such as metformin and 5-aminoimidazole-4-carboxamide ribonucleotide (1), to not only increase muscle glucose uptake, thus rendering a glucose-lowering effect, but also exert therapeutical impacts on obesity, inflammation, and cancer (31, 32, 33).

We acknowledge that there exist several limitations in this study. First, the sample size of animal models in PET–CT studies on muscle glucose uptake was small, which accounts for not achieving statistical significances in these assays. Second, there is a disconnect in muscle cell lines employed in this study because of difficulty in tracing GLUT4 translocation and glucose uptake into natural cell line—C2C12 cell. Therefore, we utilized rat myoblast cell line L6 overexpressing GLUT4—L6 GLUT4myc cell, as a substitute to study the effect of scEMC10 on glucose uptake and GLUT4 translocation. Third, the molecular mechanism whereby scEMC10 modulates AMPK and insulin signaling pathways remains elusive, which warrants further exploration.

In sum, we present evidence that scEMC10 inhibits muscle glucose uptake *via* suppression of GLUT4. Manipulation of both GLUT4 expression and translocation *via* AMPK and insulin signaling pathways is largely responsible for the scEMC10-modulated glucose uptake. The increased glucose uptake into muscle exerted by an scEMC10-neutralizing antibody suggests scEMC10 as a tractable therapeutic target for type 2 diabetes.

## Experimental procedures

### Cell culture

Both rat myoblast cell line stably overexpressing GLUT4 (L6-GLUT4myc) (34) and C2C12 myoblasts were kindly provided by Dr Qinghua Wang (Department of Endocrinology, Huashan Hospital, Fudan University, Shanghai, China) and authenticated by the supplier. L6-GLUT4myc myoblasts were cultured in minimal essential medium-α supplemented with 10% fetal bovine serum (FBS) and 1 U/ml penicillin–streptomycin and then switched to differentiation medium containing 2% FBS for 6 days when the cells reached 70% to 80% confluence. C2C12 myoblasts were maintained in Dulbecco's modified Eagle's medium supplemented with 10% FBS and 1 U/ml penicillin–streptomycin, and their differentiation, which usually took 6 to 8 days, was induced by switch to medium containing 2% heat-inactivated horse serum when myocytes were ∼80% confluent.

### Glucose uptake

Differentiated L6-GLUT4myc myotubes were exposed to recombinant mouse scEMC10 protein conjugated with human IgG1 Fc (PZ0104; Phrenzer Biotechnology), insulin, or their combination at various concentrations for different periods in the presence of 100 μmol/l 2-NBDG (N13195; Thermo Fisher Scientific) at 37 °C. Then the cells were digested and centrifuged, and the pellets were washed twice with cold PBS. 2-NBDG uptake was measured at the FITC channel (excitation/emission = 465/540 nm) by flow cytometer (Beckman).

### GLUT4 translocation assay

L6-GLUT4myc myoblasts grown on slides were treated with 1 μg/ml recombinant scEMC10 with or without 4 μg/ml monoclonal anti-scEMC10 antibody (4C2) (PZ0103; Phrenzer Biotechnology) for 30 min followed by stimulation with 100 nmol/l insulin for 30 min. It is important that before treating the cells, scEMC10 should be incubated with anti-scEMC10 antibody in a 37 °C water bath for 2 h to facilitate their binding. Then the cells were fixed, nonpermeabilized, or permeabilized with 0.1% (v/v) Triton X-100, blocked with 10% goat serum (Sango Biotech), and incubated with GLUT4 primary antibody (66846-1-Ig; Proteintech) overnight at 4 °C. On the next day, the cells were incubated with fluorescent secondary antibody (Recordbio Biotechnology) and FITC phalloidin (CA1620; Solarbio) for 1 h at room temperature followed by an additional 10-min incubation with 4′,6-diamidino-2-phenylindole (4083; Cell Signaling Technology). The fluorescent images were captured with a fluorescence microscope (Leica).

### PET–CT scan

After overnight starvation, mice were injected intraperitoneally with ^18^F-fluorodeoxyglucose (10 mCi per kg body weight) 1 h before PET–CT scan (Siemens Inveon microPET). PET–CT images were reconstructed using the ordered subset expectation maximization (OSEM3D) with the following parameters: two iterations, image zoom 1, matrix size of 128 × 128 × 159 with a voxel size of 0.815 mm × 0.815 mm × 0.796 mm. Three-dimensional ellipsoidal regions of interest with a size of 10 mm^3^ were manually drawn over the gastrocnemius muscle on the fused PET–CT images. Using the Inveon Research Workplace 3.0 software, the tracer uptake was measured, and the standard uptake values for each regions of interest were calculated to assess the glucose uptake into the gastrocnemius muscle. Standard uptake values normalize radiotracer uptake to the injected dose and animal body weight.

### Animal experiments

Mice were kept under a 12-h light–dark cycle at constant humidity (45%–65%) and temperature (20–24 °C) with free access to rodent pellet food and water. All animal studies were approved by the Institutional Animal Care and Use Committee of Fudan University.

*Emc10* gene KO mice were constructed as previously described (13). Male *Emc10* KO mice and their WT littermates aged 6 to 7 weeks were fed an HFD (D12492; Research Diets) for 8 weeks. Post overnight starvation, mice were performed PET–CT scans to assess glucose uptake into gastrocnemius and then euthanized for collecting gastrocnemius tissue for further analysis.

C57BL/6J male mice at the age of 7 to 8 weeks were starved overnight and then injected intraperitoneally with recombinant mouse scEMC10 at the concentration of 6 mg per kg body weight or vehicle (human IgG1 Fc, which is a tag conjugated with recombinant mouse scEMC10 for facilitation of purification of the scEMC10 protein) for 30 min to investigate the role of scEMC10 in the regulation of muscle AMPK signaling by Western blot, for 1 h to assess glucose uptake into gastrocnemius by PET–CT scan, or for 7 h to measure GLUT4 protein levels in gastrocnemius by Western blot.

For the antibody neutralization experiments, C57BL/6J male mice at the age of 6 to 7 weeks were fed an HFD for 8 weeks followed by intraperitoneal injection of monoclonal anti-scEMC10 antibody 4C2 at the concentration of 3 mg per kg body weight or control IgG twice a week for 2 weeks, simultaneously with the HFD. After being starved overnight, these mice underwent PET–CT scans to assess glucose uptake into the gastrocnemius and then were euthanized to collect gastrocnemius tissue for gene/protein expression or immunofluorescent analyses.

### Immunofluorescence assay

Paraffin-embedded gastrocnemius slices were heated in an oven at 65 °C for 1 to 2 h and then deparaffinized by sequential immersion in the following chemicals: xylene (I) for 60 min, xylene (II) for 5 min, 100% alcohol for 5 min, 90% alcohol for 5 min, 80% alcohol for 5 min, and 70% alcohol for 5 min. The slices were then washed three times with PBS (pH 7.4) and immersed in preheated EDTA antigen retrieval buffer (Recordbio Biotechnology). After natural cooling, the slices were washed three times with PBS and then blocked with 10% goat serum for 30 min at room temperature, followed by incubation with anti-GLUT4 (1:200 dilution, 66846-1-Ig; Proteintech) and antidystrophin (1:200 dilution, 12715-1-AP; Proteintech) antibodies overnight at 4 °C. Primary antibodies were removed by washing three times with PBS, and then the slices were incubated with horseradish peroxidase–labeled secondary antibody (Recordbio Biotechnology) at room temperature for 1 h. The slices were stained with 4′,6-diamidino-2-phenylindole and then covered with coverslips. Images were captured using a fluorescence microscope (Leica).

### RNA extraction and quantitative real-time PCR

Total RNA was extracted with TRIzol Reagent (Invitrogen) and reverse transcribed to complementary DNA using the cDNA Synthesis Kit (YEASEN). Quantitative real-time PCRs were performed according to the manufacturer’s protocols (YEASEN). The reactions were carried out using QuantStudio 6 system (Thermo Fisher Scientific) with the following conditions: 5 min at 95 °C, 40 cycles consisting of 10 s at 95 °C, 20 s at 60 °C, and 20 s at 72 °C. Gene expression was determined using the comparative 2-ΔΔCt method, normalized to *Gapdh* expression, and expressed as fold change. Primer sequences are listed in Table S1.

### Protein extraction and Western blot

Cells and tissues were lysed in radioimmunoprecipitation assay buffer, and protein concentrations were measured using the BCA Protein Assay kit (Thermo Fisher Scientific). For Western blotting analysis, 20 to 50 μg protein lysates were run in SDS-PAGE, transferred to a polyvinylidene fluoride membrane, and then blotted overnight with specific primary antibodies at 4 °C followed by incubation with appropriate secondary antibodies at room temperature for 1 h. The dilutions of primary antibodies are listed as follows: GLUT4 (1:1000 dilution, 66846-1-Ig; Proteintech), p-AMPK (1:1000 dilution, 2535T; Cell Signaling Technology), AMPK (1:1000 dilution, 5831T; Cell Signaling Technology), p-ACC (1:1000 dilution, 11818T; Cell Signaling Technology), ACC (1:1000 dilution, 3676T; Cell Signaling Technology), p-TBC1D1 (1:1000 dilution, PA5-104666; Thermo Fisher Scientific), TBC1D1 (1:1000 dilution, 22124-1-AP; Proteintech), p-AKT (1:1000 dilution, 9271S; Cell Signaling Technology), AKT (1:1000 dilution, 9272; Cell Signaling Technology), and HSP90 (1:10,000 dilution, 60318-1-Ig; Proteintech). All antibodies used were commercially sourced and were selected based on the validation data provided by the manufacturers. Blots were developed using a gel imager (Guang Yi Company Limited), and grayscale values of bands were measured using ImageJ software (National Institutes of Health).

### Statistical analysis

All statistical analyses were carried out using GraphPad Prism 8.4.3 (GraphPad Software, Inc). Data were presented as the means ± SD. Unpaired two-tailed Student's *t* test was performed to calculate differences between groups as appropriate. *p* < 0.05 was considered statistically significant.

## Prior presentation

Some of these results have been presented in abstract form at the 83rd Scientific Sessions of the American Diabetes Association, June 23 to 26, 2023 in San Diego, CA (abstract number: 1631-P).

## Data availability

The data that support the findings of this study are available upon request from the corresponding author.

## Supporting information

This article contains supporting information.

## Conflict of interest

M.B. received honoraria as a consultant and speaker from Amgen, AstraZeneca, Bayer, Boehringer-Ingelheim, Lilly, Novo Nordisk, Novartis, Pfizer, and Sanofi. All the other authors declare that they have no conflicts of interest with the contents of this article.
