# Supplementary material for: Secreted EMC10 inhibits muscle GLUT4 activity and glucose uptake in mice
Source: J Biol Chem. 2025 May 27;301(7):110296. doi: 10.1016/j.jbc.2025.110296 (PMC12221355; doi:10.1016/j.jbc.2025.110296)
Supplement: Supplemental Data [file mmc1.docx]

**
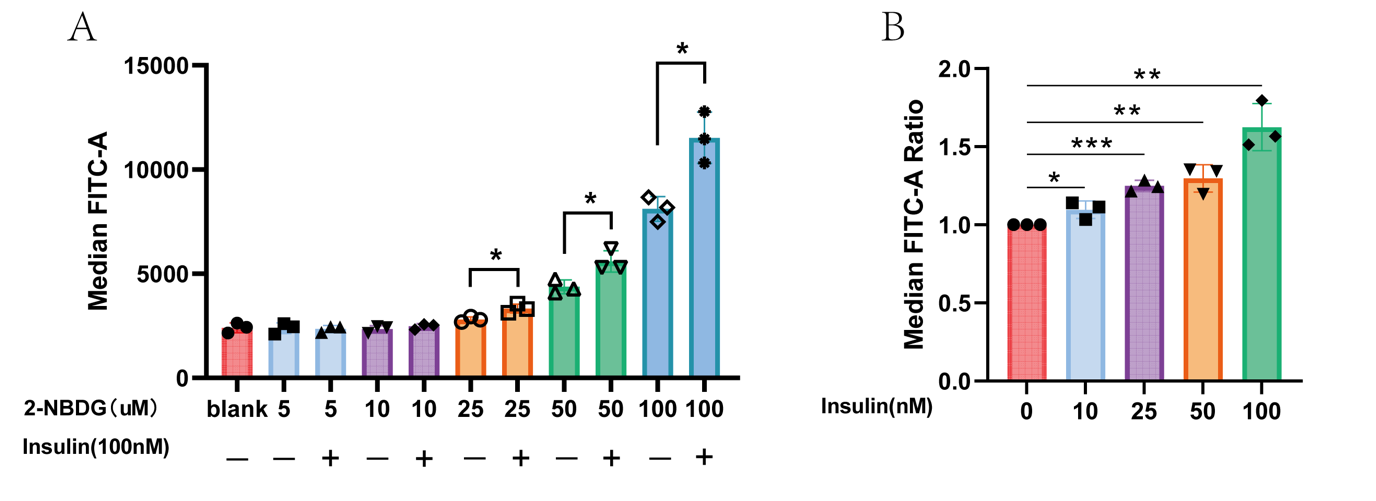
**

**Supplementary Figure 1-Insulin increases glucose uptake into L6-GLUT4myc myoblasts.** *A:* L6-GLUT4myc myoblasts were incubated with 2-NBDG at various concentrations as indicated with or without 100 nM insulin for 30 minutes, and then 2-NBDG uptake into the myoblasts was measured at the FITC channel by flow cytometer. *B:* In the presence of 100 µmol/L 2-NBDG, L6-GLUT4myc myoblasts were stimulated with insulin at different concentrations as indicated for 30 minutes, and then 2-NBDG uptake into the myoblasts was measured by flow cytometer. All cell culture experiments were repeated 3 times. All data are presented with means ± SEM. In experiments where samples were prepared in several batches, the control conditions were set at 100% in individual experiments and therefore had no error estimates. Statistical analyses were performed using unpaired two-tailed Student's *t*-test and significant differences were indicated with *P* values. **P* < 0.05, ***P* < 0.01, ****P* < 0.001.

**Supplementary Table 1. Primer sequences used in qPCR experiments of this study**

| Gene | Forward primer | Reverse primer |
| --- | --- | --- |
| GLUT4 | GTGACTGGAACACTGGTCCTA | CCAGCCACGTTGCATTGTAG |
| HDAC5 | TGCAGCACGTTTTGCTCCT | GACAGCTCCCCAGTTTTGGT |
| MEF2D | CGAGATCGCGCTCATCATCTT | AGCCGTTGAAACCCTTCTTCC |
| KLF15 | GAGACCTTCTCGTCACCGAAA | GCTGGAGACATCGCTGTCAT |
| PGC-1α | AAGTGGTGTAGCGACCAATCG | AATGAGGGCAATCCGTCTTCA |
| MyoD | CCACTCCGGGACATAGACTTG | AAAAGCGCAGGTCTGGTGAG |
| HKII | TGATCGCCTGCTTATTCACGG | AACCGCCTAGAAATCTCCAGA |
| PFK2 | GACAAGCCAACTCACAACTTCC | ACACTGTAATTTCTTGGACGCC |
| Pdha1 | GAAATGTGACCTTCATCGGCT | GAAATGTGACCTTCATCGGCT |
| PDK4 | AGGGAGGTCGAGCTGTTCTC | GGAGTGTTCACTAAGCGGTCA |
| Nr4a1 | TTGAGTTCGGCAAGCCTACC | GTGTACCCGTCCATGAAGGTG |
| Nr4a3 | AGGATTCACTGATCTCCCCAA | GATGCAGGACAAGTCCATTGC |
| GAPDH | AGGTCGGTGTGAACGGATTTG | TGTAGACCATGTAGTTGAGGTCA |
